# Imaging Genetic Based Mediation Analysis for Human Cognition

**DOI:** 10.3389/fnins.2022.824069

**Published:** 2022-04-28

**Authors:** Tingan Chen, Abhishek Mandal, Hongtu Zhu, Rongjie Liu

**Affiliations:** ^1^Department of Statistics, Florida State University, Tallahassee, FL, United States; ^2^Department of Biostatistics, University of North Carolina at Chapel Hill, Chapel Hill, NC, United States

**Keywords:** imaging genetic, human brain connectome, mediation analysis, causal inference, human cognition

## Abstract

The brain connectome maps the structural and functional connectivity that forms an important neurobiological basis for the analysis of human cognitive traits while the genetic predisposition and our cognition ability are frequently found in close association. The issue of how genetic architecture and brain connectome causally affect human behaviors remains unknown. To seek for the potential causal relationship, in this paper, we carried out the causal pathway analysis from single nucleotide polymorphism (SNP) data to four common human cognitive traits, mediated by the brain connectome. Specifically, we selected 942 SNPs that are significantly associated with the brain connectome, and then estimated the direct and indirect effect on the human traits for each SNP. We found out that a majority of the selected SNPs have significant direct effects on human traits and discussed the trait-related brain regions and their implications.

## 1. Introduction

Advances in genetics help identify the genetic contribution to human cognition and understand the influence of specific genetic variants on cognition. The analysis of genetic variations, e.g., single nucleotide polymorphisms (SNPs), is to thoroughly understand phenotypic characteristics and the genetic mechanism of both normal and disordered brain function and behavior (Shen and Thompson, 2019). However, as the cognitive phenotypes are usually self-reported and not explicit, potentially weakening the genetic effect (Fossella et al., 2002; Goldberg and Weinberger, 2004; Green et al., 2008), the mechanisms regarding how genetic variations affect such cognitive phenotypes are not clear yet (Bi et al., 2017).

To bridge the gap between genetic variations and cognitive phenotypes, neuroimaging genetics has attracted dramatic attention (Elliott et al., 2018). In particular, with the rapid development of brain imaging acquisition techniques, a number of large-scale imaging genetic databases have been established, including the Human Connectome project (HCP) study (Van Essen et al., 2013), the UK biobank (UKbb) study (Miller et al., 2016), and the Alzheimer's Disease Neuroimaging Initiative (ADNI) study (Mueller et al., 2005), among many others. By utilizing the multi-scale data available in these imaging genetic databases, genetic variations are integrated with multimodal brain imaging, coupled with clinical and environmental factors to investigate how genes are expressed through the neuroimaging based measures and identify genetic contributors to brain activities and structures associated with cognition or neurological disorders (Nathoo et al., 2019).

Recently, some neuroimaging genetics studies have been witnessed focusing on the intersection of cognitive neuroscience and behavioral genetics (Green et al., 2008, 2013; Bi et al., 2017; Luo et al., 2018), in which the goal is to specify the pathway that genetic effects on cognitive phenotypes are mediated by specific brain functions (Green et al., 2013). To achieve the goal, some statistical analysis approaches have been developed, typically consisting of two key components: (i) genome-wide association studies (GWAS) to identify genetic variants that have specific effects on specific brain areas; and (ii) mediation analysis to test if those brain areas produce a cognitive function (Green et al., 2013). Guen et al. (2018) explored the effect of genes on cognitive ability and found that brain activation patterns for cognitive traits can be genetic and also some regions' activation in the brain and cognitive abilities share the same genetic characteristics. He et al. (2021) proposed the possible linkage and relationship between genetic variants, brain morphometry, and working memory performance and found an SNP which might be influential on working memory. However, few works have been conducted to test the neuroimaging mediation in terms of the brain connectome although the study of brain networks becomes increasingly significant (Fornito et al., 2015; Lynn and Bassett, 2019). The main challenge comes from the construction of efficient representations for the brain connectome (Park and Friston, 2013). Typically, the functional connectivity can be measured from the statistical dependencies between physiological measures of brain activity (Van Den Heuvel and Pol, 2010) while the structural connectivity can be measured through diffusion tractography (Kazumata et al., 2019). However, the constructed connectivity graph is critically sensitive to the choice of brain atlas, including the number of region of interest (ROI) and their definitions (Balsters et al., 2016). Furthermore, unlike the voxel-wised or region-based neuroimaging phenotypes, it is difficult to conduct the GWAS for brain connectivity graph-based phenotypes to identify the corresponding potential genetic variants (Nathoo et al., 2019).

To address these issues, in this paper, we develop an imaging genetic-based mediation analysis framework to investigate the genetic effects on human cognition phenotypes mediated by an efficient representation of the brain connectome. Specifically, we focus on the HCP neuroimaging genetic study which facilitated many advancements in the field of brain networks (Craddock et al., 2015). To build up the representation of the brain connectome, an unsupervised statistical learning approach is proposed based on the tractography results extracted from the diffusion magnetic resonance imaging (MRI) data. GWAS are conducted for each brain connectome phenotype to detect the significant SNP-connectome pairs. For selected causal SNP, a regression analysis is conducted to test the mediation of the tied brain connectome *via* checking if it significantly affects the cognitive phenotypes.

The proposed method brings three contributions. First, a data-driven based representation of the brain connectome is developed, which possesses potential power in human cognition prediction. Second, the efficient representation can help detect genetic signals that are neglected. Third, the proposed framework can also be applied to other neurological disorders related to neuroimaging genetic databases, e.g., ADNI study, to understand the corresponding gene-connectome-cognition pathway. In conclusion, we provide a framework to study the casual pathway from SNPs data to cognitive traits, with the brain connectome as the mediator. Our result will identify significant mediation pathways, which can shed light on future investigation of SNPs → connectome → cognition mechanics. We will suggest SNP candidates that can affect human cognitive ability through altering brain structure, which will benefit further in future studies. The structure of the paper is as follows. In Section 2, we will describe the construction of the human brain connectome and the method used for GWAS and the linear modeling of effects. In Section 3, we will describe the analysis performed on the MR dataset and genetic dataset. In Section 4, the results derived from the above analysis will be discussed. Further discussions on the results and conclusion will be included in Section 5.

## 2. Method

In this section, we initially develop the human brain connectome based on fiber clustering on the diffusion MRI image data. After developing the brain connectome, we perform GWAS based on the structure connectivity representation to first reduce the dimension of the genetic data and then detect a significant relationship between SNPs and brain connectome. After the selection of the significant SNPs with their paired regions, we try to explore the relationship between human cognitive traits and SNPs mediated by the brain connectome.

### 2.1. Image Based Human Brain Connectome

#### 2.1.1. Preprocessing

Many studies have examined parcellation in a supervised way with an existing atlas (Guevara et al., 2012; Jin et al., 2014; Gupta et al., 2017). In Liu et al. (2021), an unsupervised population-level approach is developed. Borrowing from this idea, we collect all the fibers together as a large fiber set after fiber tracking, denoted as F. Let $F=\left\{f_{ij},k=1,\ldots ,m_{i},i=1,\ldots ,n\right\}$, where *f*_*ij*_ denotes the *j*-th fiber of the *i*-th individual, *m*_*i*_ denotes the total number of fibers in *i*-th subject, and *n* denotes the number of subjects. Based on the length of each individual fiber, the number of points representation within a fiber varies from 4 to 60. Most clustering methods thus involve up/down-sampling of the fibers to uniform the number of points (Guevara et al., 2012). For simplicity, we take two end points {*a*_*ij*_} and {*b*_*ij*_} of each fiber *f*_*ij*_ as the representation. More specifically, {*a*_*ij*_} and {*b*_*ij*_} are the 3D coordinates of the two end points, respectively, which are stacked into a single 6 ×1 vector ${\left(a_{ij}^{T},b_{ij}^{T}\right)}^{T}$, with the order determined by the value of their coordinates. The fibers of the *i*-th subject {*f*_*ij*_, *j* = 1, …, *m*_*i*_} is then represented as a matrix *c*_*i*_ with size of 6 × *m*_*i*_. The *m*_*i*_ represents the number of all the fibers in *i*_*th*_ subject, which does not have to be the same across all subjects. The individual *c*_*i*_s are then collected by simply horizontally stacking the matrices *c*_*i*_s together as *C* = (*c*_1_, …, *c*_*n*_) with the dimension of $6\times \overset{n}{\underset{i=1}{\sum }}m_{i}$, which is the reduced representation of F.

#### 2.1.2. Fiber Clustering

At this end, all the fibers are represented in *C*. Since the original K-means algorithm has a high computational burden, we adopt a mini-batch K-means algorithm (Cho and An, 2014). In each iteration, a random set of fibers were selected to update the centroids, until we provide a stable partition on *C* as $A_{K}=\left\{A_{K}^{\left(1\right)},\ldots ,A_{K}^{\left(K\right)}\right\}$, where K is the number of cluster and each $A_{K}^{\left(k\right)}$ interpreted as the *k*-th cluster. In the brain connectome studies, the parcellation number *K* varies from two to sometimes tens of thousands. Currently, the choice of exact cluster number remains an open problem (Eickhoff et al., 2015). A discussion about the choice of cluster number was found in Liu et al. (2021), which provides a guideline to reduce the prediction error on some specific human traits while preserving stable connectivity features.

#### 2.1.3. Brain Connectome

After the fiber clusters are acquired, a subject-wise connectome is defined based on the distribution of the fibers across population clusters. Specifically, the connectome for the *i*-th subject is defined as a *K* dimension vector $\omega_{i}$, where *K* is the number of clusters. For *k* = 1, …, *K*, define ω_*ik*_ as the proportion of number of the *i*-th subject's fibers within cluster *k* to the total number of fibers of the *i*-th subject,


$$\begin{array}{l} \omega_{ik}\\ =\frac{\text{Number of fibers of the i}-\text{th subject within the population cluster k}}{\text{Total number of fibers of the i-th subject}}. \end{array}$$

The connectome of subject *i* is then defined as $\omega_{i}=\left\{\omega_{i1},\ldots ,\omega_{iK}\right\}$. One of the interpretations about the connectome with *K* clusters is that they are distinct groups of associations pathways on the granularity level induced in our brain. With this interpretation, ω_*i*_ seeks to comprehensively capture the proportion of these individual associations in *i*-th subject's brain. In addition, it is important to notice that a larger ω_*ik*_ can have two non-exclusive implications: (i) *i*-th subject has a denser axons presence in cluster *k* compared with other clusters; and (ii) *i*-th subject has a bigger volume of the relevant pair of gray matter areas that cover more fibers. This differs from our connectome method with existing functional connection based methods.

### 2.2. GWAS Based on Connectome Representation

First, the potential causal SNPs are identified such that the dimension of the genetic data can be reduced from a very large scale to a moderate scale and then the significant connectivity-SNP pairs are detected. Let G be the set of *N*_*G*_ single nucleotide polymorphisms (SNPs). For the *i*-th subject, let $x_{i}^{g}$ take the values of 0 (no minor allele), 1 (one minor allele), and 2 (two minor alleles), indicating the genetic data at the *g*-th locus in G, *g* = 1, …, *N*_*G*_, and $Z_{i}={\left(z_{i1},\ldots ,z_{ip}\right)}^{⊤}$ be a *p*×1 vector including the clinical confounders, e.g., age and gender. To adjust for population structures, we also included the scores for the top 2 principal components as the confounders.

Based on image-based human brain connectome, assume that *K* brain parcellations are derived from the diffusion MRI scans and $\omega_{i}={\left(\omega_{i1},\ldots ,\omega_{iK}\right)}^{⊤}$ is the structure connectivity representation for the *i*-th subject, i.e., a *K*×1 vector containing the individual cluster weights satisfying ω_*ik*_≥0 and $\underset{k}{\sum }\omega_{ik}=1$. The next step is to establish a linear relationship between each SNP $x_{i}^{g}$ and connectome ω_*ik*_.


(1) $$\begin{array}{r} \omega_{ik}=\beta_{0}+x_{i}^{g}\beta_{k}+Z_{i}\gamma_{k}+\epsilon_{ik}, \end{array}$$

where *i* = 1, ⋯ , *n*, *k* = 1, ⋯ , *K*, and *g* = 1, ⋯ , *N*_*G*_. *Z*_*i*_ denotes confounders, β_0_ is intercept and β_*k*_, γ_*k*_ are the coefficients in the above equation. ϵ_*ik*_ is the error and we assume that


$$\epsilon_{ik}\sim N\left(0,\sigma_{1}^{2}\right),$$

where ϵ_*ik*_s are iid from normal distribution with constant variance $\sigma_{1}^{2}$. We have a huge number of genes out of which a small number of genes might have effect on ω_*ik*_ hence in the model not all SNPs $x_{i}^{g}$ have significant effect on ω_*ik*_. To have an initial screening, we can test the hypothesis of *H*_10_: β_*k*_ = 0 vs. *H*_11_: β_*k*_≠0. By testing the above hypothesis, we can reduce the number of relevant genes in our model and hence reduce the dimension by dropping the SNPs where the *p*-value is large for the corresponding coefficients. Dimension reduction by dropping insignificant SNPs can help us increase the computation efficiency and it also makes the estimates biased and power gets reduced.

The subjects for which both SNP data and connectome data are available have been taken into consideration for the analysis. The estimation of the parameters in regression Equation (1) was carried out in open-source software PLINK (https://www.cog-genomics.org/plink) that is the whole genome data analysis toolset and cluster-wise results were derived containing the estimates of the parameter β_*k*_ and its corresponding *p*-value. The selection of SNPs was made by testing the hypothesis mentioned above for parameter in Equation (1) and discarding those SNPs for which the hypothesis has been accepted. To identify significant variants, we used false discovery rate (FDR) control to select potential SNPs from our GWAS results. In this step, 942 SNPs were selected controlling for an expected FDR of 0.05, and the FDR controls were done for each cluster separately. Since the SNPs selected in this way can have a correlation with each other, we also fed our GWAS result into FUMA (Watanabe et al., 2017a) to identify independent lead SNPs with a measure of Linkage disequilibrium *r*^2^ ≤ 0.1. The top SNPs that appear both in our FDR-selected SNPs list and in the lead SNPs of FUMA are reported in Table 1.

**Table 1:** Visualization of the four clusters and their corresponding top selected single nucleotide polymorphisms (SNPs).

**Cluster**	**SNP**	
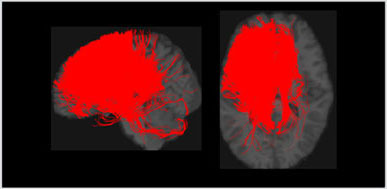	rs150026771 rs28688016 rs59894521 rs76385638 rs74606713	rs17071497 rs1990985 rs76347993 rs146800096 rs75101611
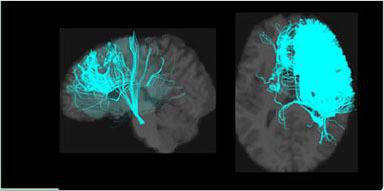	rs12427819 rs4888388 rs7880476 rs184215464 rs17128467	rs76250915 rs9727148 rs72814539 rs72857725 rs60716510
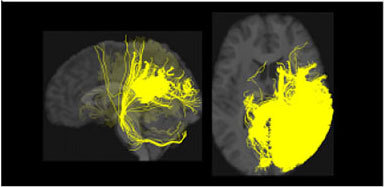	rs116202570 rs138291276 rs149212121 rs117384094 rs190073949	rs117711504 rs74606713 rs17071497 rs2494732 rs11914543
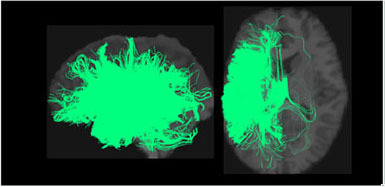	rs150123805 rs145884040 rs114047728 rs5921578 rs28564459	rs115117038 rs73723635 rs568099192 rs115436176 rs147366834

### 2.3. Genome-Wide Linear Modeling of Effects on Human Traits

In the above subsection, we have selected SNPs based on different brain regions ω_*ik*_. By matching the correspondence, we can find the related brain region with respect to each SNP. For example, for subject *i* = 1, suppose we find pairs of ω_11_ with *x*_11_, *x*_12_; ω_12_ with *x*_12_, *x*_13_; and ω_13_ with *x*_14_, *x*_11_, then we can get a conclusion of the pairs of *x*_11_ with ω_11_, ω_13_; *x*_12_ with ω_11_, ω_12_; *x*_13_ with ω_12_; and *x*_14_ with ω_13_. Thus for each significant SNP $x_{i}^{g}$, $g^{*}$ and $g^{*}$, we have its corresponding active connectome label set *S*^*g*^⊂{1, 2, ⋯ , *K*}, which will be used in the model in the next step. We adopted the additive minor allele coding to transform SNPs' information into discrete covariates. For example, if the minor allele is G, then the SNP “AA” would be encoded as 0; similarly, SNPs “AG” and “GG” would be coded as 1 and 2. We used PLINK's built in functionality to achieve this and carried out subsequent analysis.

**Figure 1:**
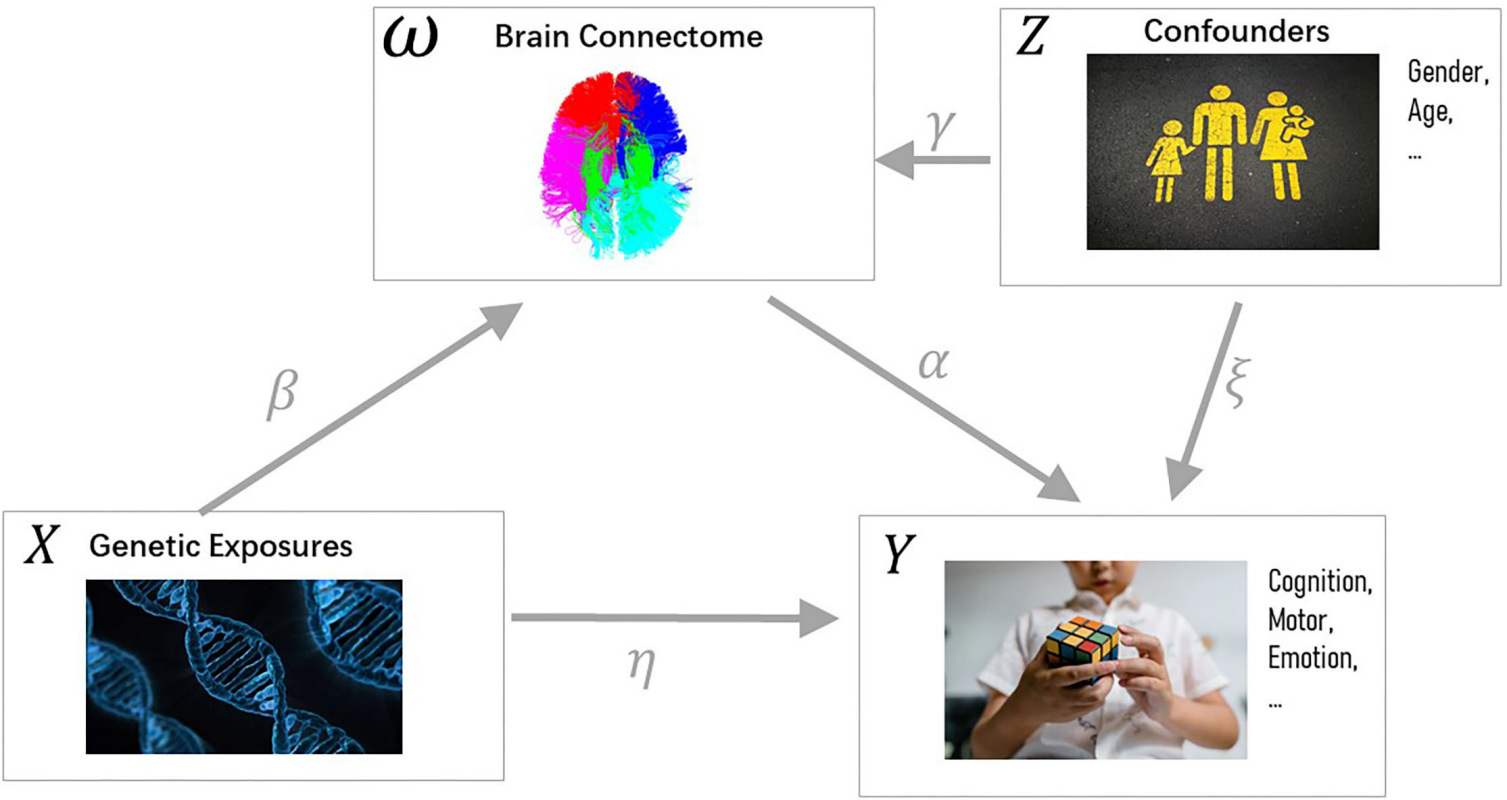
Casual pathway diagram. Variables are colored black while parameters are colored gray. The independent variable *X* denotes the genetic exposure. The dependent variable *Y* is the cognitive trait. The brain connectomes ω act as the mediator, which transmits the indirect effect of *X* to *Y*. Both ω and *Y* are affected by confounders *Z* (e.g., gender, age). Source corresponding to letter Z, top-right: https://unsplash.com/photos/KhStXRVhfog; source corresponding to letter X, bottom-left: https://pixabay.com/illustrations/dna-biology-medicine-gene-163466/; Source corresponding to letter Y, bottom-right: https://www.pexels.com/photo/close-up-photo-of-a-child-solving-a-rubik-s-cube-8471912/.

As shown in Figure 1, the brain connectome is considered a mediator in our model, which transmits the effect of genetic exposures to human traits (Mackinnon et al., 2007). To complete the rest of the model, we build a linear relationship between human traits *Y*, SNPs *X*, brain connectome ω, and confounder *Z*. Here, the regression is based on paired SNPs $x_{i}^{g}$ and brain connectome ω_*ik*_. Brain structure-related confounding factors *Z*_*i*_: gender and age are also assumed to have an direct impact on human traits.

For the *i*-th subject, the genome connectome linear model for human trait is


(2) $$\begin{array}{r} Y_{i}=\eta_{0}+x_{i}^{g}\eta +\underset{k\in S^{g}}{\sum }\omega_{ik}\alpha_{k}+Z_{i}\xi +\epsilon_{i}, \end{array}$$

where *i* = 1, ⋯ , *n*, *k* = 1, ⋯ , *K*, and $g^{*}$ . *Z*_*i*_ denotes confounders and the α_*k*_ are the coefficients for the active connectome ω_*ik*_. η_0_ is the intercept, η and ξ are the parameters while ϵ_*i*_ is the error in the above equation with the assumption that


$$\epsilon_{i}\sim N\left(0,\sigma_{2}^{2}\right),$$

where ϵ_*i*_s are iid from a normal distribution with constant variance $\sigma_{2}^{2}$. We estimate the parameters in the above Equation (2) by minimizing the error sum of squares. In Equation (2), we test the hypothesis *H*_0_:η = 0 vs. *H*_1_:η≠0 to check whether the parameter η is significant or not and we get the *p*-value of the estimate of η. For the significance of indirect pathway, we test the hypothesis *H*_10_:β_*k*_ = 0 vs. *H*_11_:β_*k*_≠0 in Equation (1) and *H*_20_:α_*k*_ = 0 vs. *H*_21_:α_*k*_≠0 in Equation (2). The adjusted *p*-values by FDR correction are obtained with the corresponding point estimates of those parameters.

## 3. Data Analysis

In this paper, we used the subjects from the HCP 1200 Subjects Release (S1200) (https://www.humanconnectome.org/storage/app/media/documentation/s1200/HCP_S1200_Release_Reference_Manual.pdf) that have all four categories of data (genetic exposures, diffusion MRI, confounders (gender and age), and cognitive traits). We used the diffusion MRI to build up the population-wise brain connectome, and combine it with the rest of the data to carry out the mediation analysis. The HCP data contains 298 (149 pairs) of genetically confirmed MZ (Monozygotic) twins and 188 subjects (94 pairs) of genetically confirmed DZ (dizygotic) twins. The GWAS studies would drop one of the MZ twins (Lowe et al., 2009; Parsons et al., 2013) to prevent such twin structure to increase the type I error rate. We followed this practice and kept only one randomly chosen sample from the MZ twins in our dataset. After matching the ID with imaging data, we finally have 870 individuals (160 DZ individuals, 138 one-of-MZ individuals, and 572 non-twin individuals) as our total sample size.

### 3.1. MR Image Dataset

The MR and behavioral dataset used in this paper is from the HCP 1200 Subjects Release. This dataset is comprised of 1,206 behavioral and MRI data from 1,206 healthy young adult participants, collected from August 2012 to October 2015. The MR data includes structural MRI, task functional Magnetic Resonance Imaging (fMRI), resting state fMRI, and diffusion Magnetic Resonance Imaging (dMRI). We focused on the diffusion MRI data and the behavioral data. The behavioral dataset contains unit tests targeting varies domains related to human behavior, which relates to alertness, cognition, emotion, motor, personality, psychiatric, and life function. A majority of these tests are developed from the (National Institutes of Health) NIH health toolbox. It can be referred to the release manual of the S1200 dataset (https://www.humanconnectome.org/study/hcp-young-adult/document/1200-subjects-data-release) and the NIH toolbox's website (https://www.healthmeasures.net/explore-measurement-systems/nih-toolbox) for the details.

### 3.2. Genetic Dataset

The genetic dataset (https://www.ncbi.nlm.nih.gov/projects/gap/cgi-bin/study.cgi?study_id=phs001364.v1.p1) from HCP was used for our analysis. In this paper, some data quality control operations were performed on the data: (1) subjects where more than 10% of the genotypes missing were removed; (2) variants where the missing genotype rate is greater than 10% were also removed from the data; and (3) variants that failed the Hardy-Weinberg test at the 10^−7^ level of significance were also removed. Finally, a total of 2062590 SNPs are obtained after the quality control.

## 4. Results

### 4.1. GWAS and Paired Brain Connectome

In our GWAS analysis, a total of 942 SNPs have been found significantly associated with 5 out of the 10 clusters (c1,c3,c4,c7,c8). In Table 1, the top SNPs with the lowest *p*-values for the major four clusters are shown. The clusters and brain images belong to a randomly selected subject and the clusters are colored arbitrarily to help distinguish them. As mentioned in Section 2.2, these top SNPs are independent of each other at *r*^2^ ≤ 0.1.

**Figure 2:**
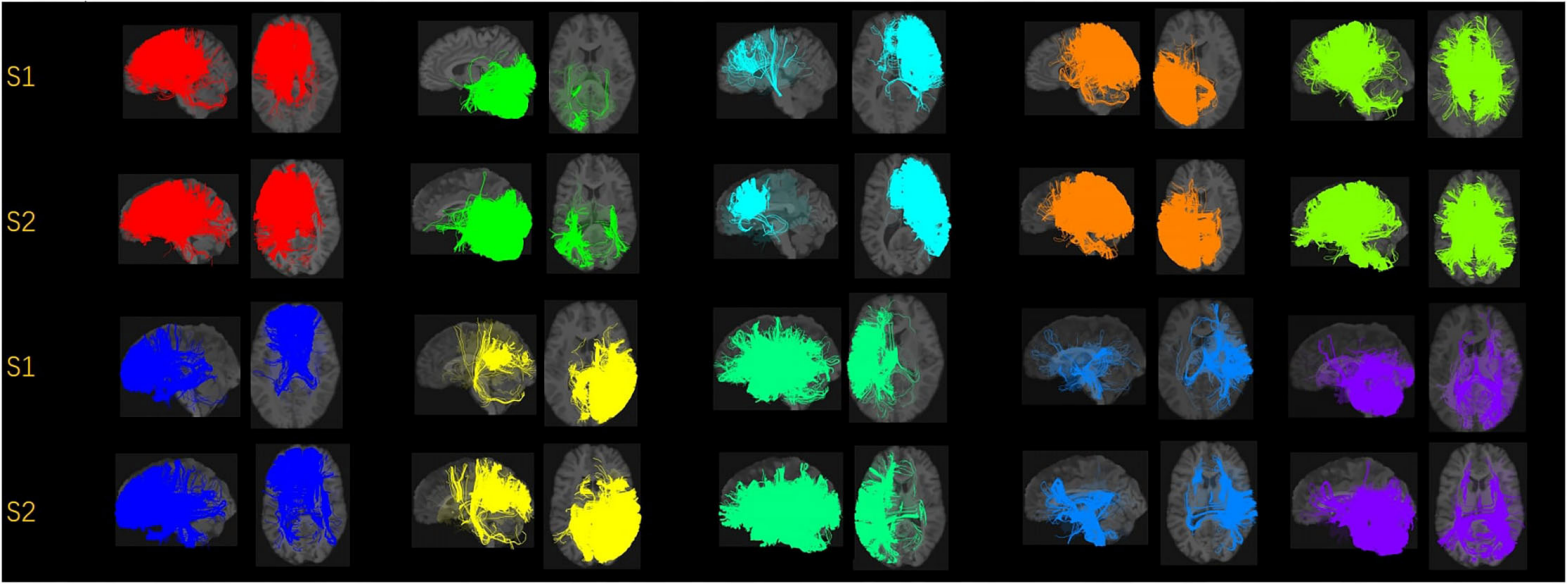
Visualization of the fiber clusters of two randomly chosen subjects. Rows 1 and 3 show clusters 1 through 10 for subject S1, while rows 2 and 4 show clusters 1 through 10 for subject S2. It shows the heterogeneity of their brains and connectomes.

In Figure 2, we displayed the 10 clusters of two different subjects, with each row corresponding to each subject. The overall regions for those clusters are similar but exist heterogeneity in different subjects. The different shapes of the white matter show that the second subject in the second row has a slightly skinnier skull. Also, the second subject has slightly less amount of fibers distributed in the second (green) cluster, compared to other clusters. It is worth noticing that in the definition of ω_*ik*_ (Section 2.1.3), the denominator is the total number of fibers of the *i*-th subject. Thus, we are more interested in the individual's distribution of fibers, rather than the absolute number of fibers.

To help facilitate the understanding of the potential causal effect of the SNPs, we also used the Functional Mapping and Annotation (FUMA) for functional annotation (Watanabe et al., 2017b). We included SNPs in LD with our SNPs from the 1,000 Genomes reference panel provided by FUMA (https://fuma.ctglab.nl). We reported the functional annotation for the top SNPs corresponding to each cluster in Supplementary Table 1. We also used FUMA's gene-based test tool MAGMA (de Leeuw et al., 2015) to identify significant genes.

### 4.2. Causal Pathway Analysis

Mediating variables are used to transmit the effect of an independent variable into the response variable. Mediation analysis is a prominent method to estimate causal relationship, which is a way to understand the effect of a third variable on the relationship between two variables. Here, we focus on the effect of the human brain connectome on the relationship between human traits and SNPs. With an aim to estimate the average direct effect (ADE) of SNPs on human cognition ability, we test the hypothesis *H*_0_:η = 0 vs. *H*_1_:η≠0 for the parameter η in the Equation (2). We consider the *p*-values and if the *p*-values are less than 0.05, then we reject the null hypothesis of no direct effect at 95% level of confidence. If the null hypothesis is rejected for an SNP, then it indicates that this SNP has a direct effect on human cognition.

Now, we have selected those SNPs that have a significant effect on clusters of the human brain connectome (refer to Equation (1)). Hence, the parameter corresponding to ω_*ik*_ in Equation (2) together with the parameter β_*k*_ from Equation (1) determines the extent of an indirect effect of the gene on human traits. One way to estimate the significance of the indirect effect is through testing hypothesis. Testing the hypothesis *H*_20_:α_*k*_ = 0 vs. *H*_21_:α_*k*_≠0 and *H*_30_:α_*k*_β_*k*_ = 0 vs. *H*_31_:α_*k*_β_*k*_≠0 would determine the significance of indirect effect of each selected SNP on human trait.

**Figure 3:**
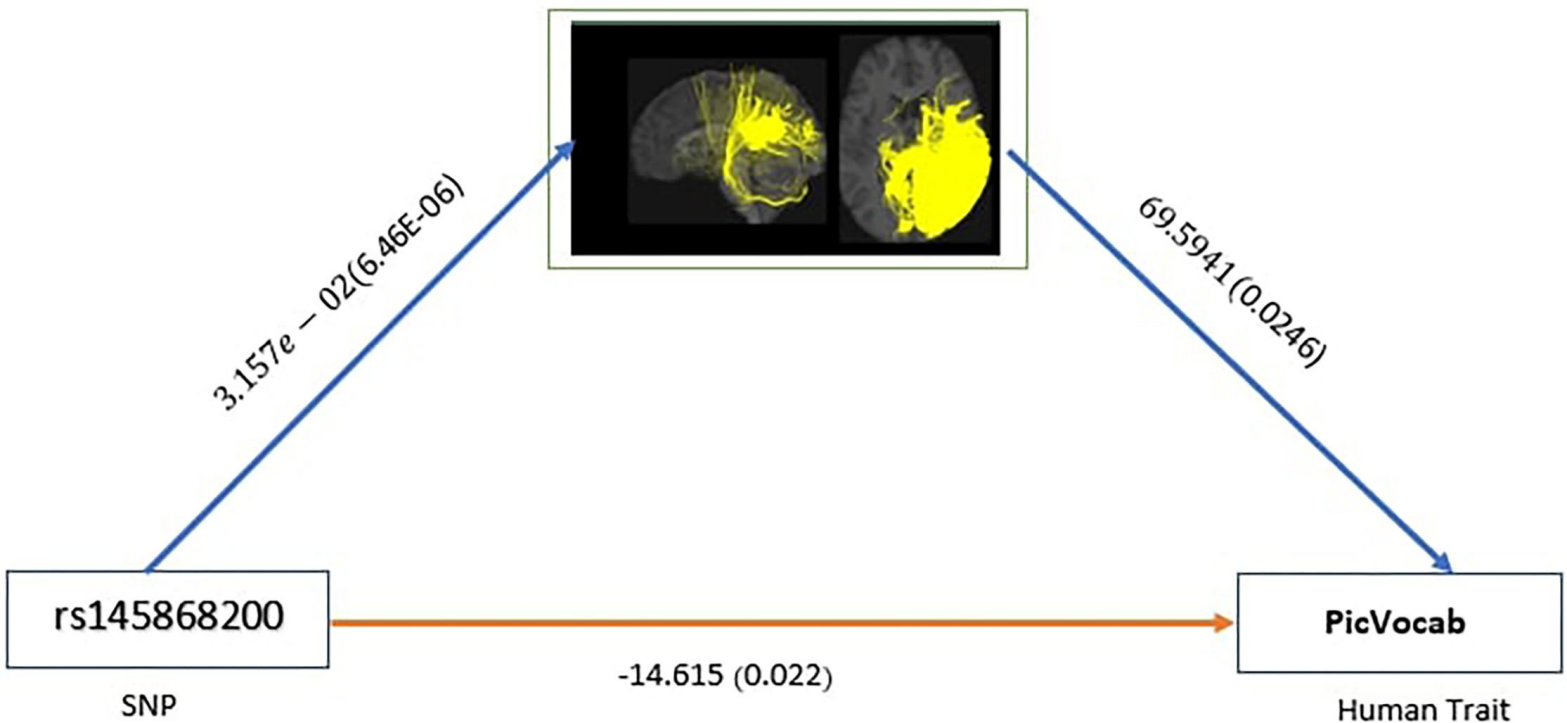
AIE for SNP rs145868200 with significant ADE with *p*-value <0.05 showing mediation pathways through connectome.

For our model, we have considered four human traits, i.e., oral reading score (ReadEng), list sorting score (ListSort), card sort score (CardSort), and receptive vocabulary score (PicVocab). To estimate the average indirect effect (AIE) for SNPs, we consider the parameter β_*k*_ estimated from Equation (1) and α estimated from Equation (2) and add the product of the coefficients ($\underset{k\in S^{g}}{\sum }\alpha_{k}\beta_{k}$) to get an overall indirect effect. For each of the four traits, we examined the effects of the SNPs selected based on the *p*-value of the ADE. As shown in Table 2, we also show the percentages of the intermediate effect calculated by: $\frac{abs\left(AIE\right)}{abs\left(ADE\right)+abs\left(AIE\right)}$ in percentage as mentioned by Mackinnon et al. (2007). Alwin and Hauser (1975) discussed that there might be situations where the coefficients of direct effect and indirect effect will have opposite signs and hence they will counteract each other. In that scenario, ratio of indirect effect to total effect might be negative or greater than one. In this scenario, the total effect is less than the total of absolute effects and a possible solution to bypass this problem is to use the absolute value of direct effect and indirect effect. A brief summary of SNPs related to direct effect and indirect effect for the above human traits is given in Table 2. In Figure 3, an example of a significant SNP and the effect of mediation variables is shown to present the fact that the human trait PicVocab is affected by the SNP through the brain connectome.

**Table 2:** Average direct effect (ADE) and average indirect effect (AIE) are shown.

**Traits**	**SNP**	**ADE**	***P*-value**	**AIE**	**p-value of β_*k*_**	**p-value of α_*k*_**	**% of GE**
ReadEngrs	rs138291276	–8.557	1.12E-02	1.413	3.46E-07	0.015	14.169
	rs79993170	–1.861	3.26E-02	0.323	2.68E-06	0.020	14.795
	rs149111244	–12.061	1.19E-03	1.398	3.99E-06	0.016	10.39
	rs61956274	–15.969	1.14E-02	1.950	9.25E-06	0.043	10.88
	rs79993170	–2.057	6.19E-03	0.279	2.68E-06	0.020	11.93
	rs117384094	–8.339	3.48E-03	1.133	7.71E-07	0.018	11.96
PicVocab	rs113713560	–7.676	2.46E-02	1.137	1.1E-05	0.025	12.90
	rs117261313	–14.615	2.24E-02	2.197	6.47E-06	0.025	13.07
	rs145868200	–14.615	2.24E-02	2.197	6.47E-06	0.025	13.07
	rs150476682	–14.655	2.20E-02	2.232	6.49E-06	0.022	13.22
	rs139895115	–14.641	2.20E-02	2.248	6.63E-06	0.021	13.31
	rs138291276	–6.896	1.80E-02	1.156	3.46E-07	0.021	14.35

*We see that there are 2 human traits for which SNPs have significant direct effect and significant indirect effect. We see that there is one significant mediation pathway through connectome for these SNPs. The % of global effect column signifies the percentage of global effect explained by the mediation of connectome*.

## 5. Discussion

Our brain is divided into functionally specialized regions and any cognitive task is usually involved in collaboration among two or more specialized regions (Eickhoff et al., 2015). A common assumption is that the white matter tracts among these regions provide a structural basis for the communication underlying such tasks. To check the overlaps of our clusters (Ref to Figure 2) with existing anatomically meaningful fiber bundles, we surveyed a list of ROIs that have appeared in the existing literature (Friederici and Gierhan, 2013; Gupta et al., 2017; Shin et al., 2019), which include inferior longitudinal fasciculus (ILF); corpus callosum (CC); corticospinal tract (CST); uncinate fasciculus (UF); inferior fronto-occipital fasciculus (IFOF); superior longitudinal fasciculus (SLF); arcuate fasciculus (AF). Some clusters have overlaps with only one of the ROIs in the list above. For example, clusters 2 and 10 contain only the ILF, while cluster 5 contains the CST. Other clusters might have overlaps with multiple ROIs. For example, clusters 3 and 5 contain CC and UF; clusters 8 and 9 contain multiple important language related ROIs such as SLF, AF, and IFOF (Friederici and Gierhan, 2013). Although not overlapping with any ROIs in the list, cluster 4 contains the majority of the fibers that originated from the left parietal lobe and occipital lobe. The parietal lobe can be divided into 4 subregions, among which there is the inferior parietal lobule. Inferior parietal lobule has been linked with various cognitive functions (Numssen et al., 2020), some of which are believed to be lateralized to the left hemisphere, for example, our language function (Friederici, 2016). Others have also observed the left inferior parietal lobule's importance in mathematical reasoning (Eliez et al., 2001) and perspective tasks (Arora et al., 2015).

The mediation analysis is of great scientific interest to explore the extent of dependence on a mediator. Here, we have performed the mediation analysis to trace the causal pathway between SNPs and human traits through the human brain connectome. The SNPs that have both significant direct effect (DE) and indirect effect (IE) were selected according to the *p*-values for both effects were smaller than 0.05. In total, we found 2 and 453 SNPs (out of 942 total SNPs) for human traits ReadEng and PicVocab, respectively. The top SNPs for ReadEng and 10 SNPs for PicVocab are reported in Table 2. In Table 2, the *p*-values of β_*k*_ (see Equation (1)) and α_*k*_ (see Equation (2)) are shown to represent significant pathways through the connectome. The pair (β_*k*_, α_*k*_) signifies a pathway through the connectome and the pair (β_*l*_, α_*l*_) signifies another pathway through the connectome. For those SNPs with significant direct effect and indirect effect, we find that a moderate percentage (8–15%) of the global effect is mediated by the brain connectome. Among the mediation paths we found in our analysis, most of the SNPs only have mediation effects through a single cluster and the mediation through cluster 7 is of most dominant. However, this may be related to the fact that cluster 7 has the most associated (606 out of the 942 selected) SNPs in our GWAS analysis. Further analysis of other imaging genetic studies with large sample size might uncover the mediation pathways through other clusters and delineate the mediation structure through multiple pathways.

To better explain the findings of our mediation analysis, we have discussed all three possible cases as outcomes of mediation analysis: significant direct effect with insignificant indirect effect, significant indirect effect with insignificant direct effect, and significant direct effect as well as indirect effect. As our focus lies on the SNPs which have significant relationship with connectome, i.e., we have already selected the SNPs where the hypothesis *H*_10_:β_*k*_ = 0 vs. *H*_11_:β_*k*_≠0 is rejected, we proceed further in the following manner. In the first case, the hypothesis *H*_0_:η = 0 vs. *H*_1_:η≠0 is rejected while we fail to reject the null hypothesis *H*_20_:α_*k*_ = 0 vs. *H*_21_:α_*k*_≠0 indicating that we have significant direct effect without significant indirect effect. In the second case, we fail to reject the hypothesis *H*_0_:η = 0 vs. *H*_1_:η≠0 while the null hypothesis *H*_20_:α_*k*_ = 0 vs. *H*_21_:α_*k*_≠0 is rejected indicating that we have an insignificant direct effect but we find the mediation pathway through the connectome to be significant. In the third case, we are able to reject both hypotheses indicating that the direct effect is significant as well as the indirect effect, i.e., the mediation pathway through connectome is significant. In other words, the total effect of *x*_*i*_, i.e., SNP is transmitted to *Y*_*i*_ through two pathways, direct pathway (direct effect is denoted by η) and indirect pathway through connectome (*x*_*i*_ → ω_*ik*_→*Y*_*i*_) whose effect is observed as α_*k*_β_*k*_ termed as indirect effect. Our main focus lies on case three, i.e., both hypotheses are rejected indicating significant direct effect and a significant indirect effect. Table 3 represents the first two cases where we see only one of the DE and IDE is significant while the full results are available in Supplementary Table 2.

**Table 3:** ADE and AIE are shown for cases where we see the significant direct effect without significant indirect effect and insignificant direct effect with significant indirect effect corresponding to the human trait ReadEng.

**Cases**	**SNP**	**Direct effect**	***P*-value of DE**	**Indirect effect**	***p*-value of beta_k**	***p*-value of alpha_k**	**% of GE**
Significant IDE without significant DE	rs138843033	–9.7122	0.0657	1.9390	2.127E-06	0.0221	16.6422
	rs117384094	–6.0722	0.0659	1.2483	7.714E-07	0.0240	17.0521
	rs149175148	–13.1205	0.0732	2.4151	1.670E-05	0.0239	15.5454
	rs149111244	–7.5596	0.0798	1.5294	3.995E-06	0.0233	16.8268
	rs113713560	–6.8519	0.0832	1.3293	1.097E-05	0.0242	16.2486
Insignificant IDE with significant DE	rs79720263	9.9466	0.0000	0.4819	1.192E-05	0.1657	4.6208
	rs11572851	6.6542	0.0004	-0.5431	6.294E-06	0.0595	7.5461
	rs116785568	–17.9289	0.0006	0.9966	2.317E-07	0.2727	5.2658
	rs113673946	6.3234	0.0012	–0.5341	1.802E-05	0.0600	7.7887
	rs9342351	2.1728	0.0042	–0.2048	1.318E-05	0.0670	8.6148

To understand the causal mediation of SNPs, we selected a candidate SNP rs138291276 from the third case, i.e., SNPs with both significant direct effects and indirect effects, as shown in Table 2, this SNP has significant mediation effects on both traits: PicVocab and ReadEng. On the one hand, we found that rs138291276 is mapped to gene RPH3A, which is involved in the stabilization of N-methyl-D-aspartate receptor (NMDARs) (Franchini et al., 2019). This proposed that rs138291276's observed direct effect (DE) might be related to its expression through RPH3A. On the other hand, we also observed a moderate mediation (~14%), which suggests an indirect pathway of this SNP on human traits. The existence of both direct and indirect pathways suggests investigation of the causal mechanics of these two paths, their potential homogeneity, heterogeneity, and interaction.

For the comprehensiveness of our discussion, we also selected a candidate SNP rs4888388, from the second case, i.e., SNPs that have a significant indirect effect but not a significant direct effect. As shown in Table 1, SNP rs4888388 (imm_16_73951649) is mapped to gene CDFP1 and associated with cluster3. In Messina et al. (2017), CDFP1 is linked with microcephaly primary hereditary (MCPH), a disease that can cause a reduction in brain size and head circumference at birth. Worst cases of MCPH often result in mild to severe mental retardation (Woods et al., 2005). This evidence suggests that gene CDFP1 may be the potential medium of the mediation pathway, from SNP rs4888388 to human connectome cluster 3, and to human cognition. As mentioned before, SNP rs4888388 has an insignificant direct effect. This may propose that most of rs4888388's ability to affect human traits, is mediated through the human connectome. Lastly, the existence of this mediation pathway within the HCP1200 dataset also provides that the CDFP1's effect on brain structure and cognition not only exists in diseased patient but also among healthy young adults.

We also tried non-parametric bootstrap to obtain the mediation effects and *p*-values. We used the “mediation” (https://cran.r-project.org/web/packages/mediation/mediation.pdf) package in R to get the bootstrapped estimates and *p*-values. We used 500 simulations with a sample size of 870 (the same as our original sample size). Then, the FDR controls are adopted to adjust *p*-values for addressing the multiple test issue. We see that the bootstrapped estimates are almost identical to our original estimates while the *p*-values support the significance of the estimates. We have also considered a 2-fold cross-validation. Specifically, we have randomly divided the data of 870 subjects into two equal groups and fitted the regression model in Equation (2) for all selected 942 SNPs. We found that for PicVocab, no SNP has a significant direct effect on both groups; for ReadEng, we have found three SNPs have a significant direct effect in both groups but an insignificant indirect effect for both groups. Since the reduced power, we cannot find significant pathways for cross-validation. In future work, an external data set with a larger size will be considered.

To investigate the effect of genetically similar subjects from the same family, we have randomly selected one subject from each family and sample size of 434 individuals were considered for further analysis. As discussed in Section 2.2, after using FDR, we selected 2,433 SNPs for further mediation pathway analysis. We found that out of 2433 SNPs, 555 SNPs were selected in our earlier analysis of 942 SNPs where 870 subjects were considered. Among the four human traits, we only found significant mediation pathways for PicVocab. Compared to previous results of 453 mediation pathways, we also found most mediation pathways (67 mediation pathways) in PicVocab. Out of these 67 mediation pathways, we found 12 SNPs which were present in the set of 555 SNPs. Two SNPs, rs79993170 (JHU_1.246357095) and rs184384228, have also been found to be significant in our previous analysis. Another SNP rs138843033 that turns out to be significant can also be seen in our previous analysis with a *p*-value of direct effect as 0.06, slightly greater than our cut-off of 0.05. Thus, we conclude that SNPs rs79993170, rs184384228, and rs138843033 are considered as valid SNP candidates for mediation pathways from a conservative point of view. We have attached the results of this analysis in Supplementary Table 3. The coefficients and *p*-values of direct effect and the *p*-values of mediation pathway are included. In the table, we have also pointed out the results of the above 3 SNPs in Supplementary Table 3, which overlap with our previous analysis. We save all the results to keep the possibility of exploring mediation pathways open.

There are a couple of limitations in the findings of our current study. First, it is challenging for our proposed framework to handle the twin/family studies because it does not take into account the bias imposed by DZ twins as they share partial genes. Although removing one of the DZ twins can avoid the correlation within twins, it is inefficient due to the reduced sample size and the loss of information contained in twins. In addition, the current GWAS result is sensitive to the sampling mechanism when randomly selecting one subject from each family. In fact, we generated another four different sampling combinations of the 434 individuals, and the number of significant SNPs were 1771, 139, 2329, and 777, respectively, which varies a lot across different sampling combinations. Therefore, it is of great importance to develop advanced statistical models and address the issues in twin/family studies. Furthermore, the results need to be replicated using large samples in future studies. Second, choosing the number of clusters is still an open problem. Although the choice of 10 clusters provides us with better performance in terms of both visualization and explainability of brain connectomes, it lacks further structural justification. Thus, it will be an interesting direction to leverage brain structure and connectivity information and develop some learning techniques in finding the optimal number of clusters in brain connectomes.

## Data Availability Statement

Publicly available datasets were analyzed in this study. This data can be found here: https://www.humanconnectome.org/study/hcp-young-adult/document/1200-subjects-data-release.

## Author Contributions

TC, AM, and RL contributed to the conception and design of the study. TC and AM performed the analysis and wrote the first draft of the manuscript. HZ contributed to manuscript polishing. RL supervised the project, developed the method and wrote the manuscript. All authors provided critical feedback and helped shape the research, analysis, and manuscript. All authors contributed to the article and approved the submitted version.

## Conflict of Interest

The authors declare that the research was conducted in the absence of any commercial or financial relationships that could be construed as a potential conflict of interest.

## Publisher's Note

All claims expressed in this article are solely those of the authors and do not necessarily represent those of their affiliated organizations, or those of the publisher, the editors and the reviewers. Any product that may be evaluated in this article, or claim that may be made by its manufacturer, is not guaranteed or endorsed by the publisher.
