# Supplementary material for: Imaging Genetic Based Mediation Analysis for Human Cognition
Source: Front Neurosci. 2022 Apr 28;16:824069. doi: 10.3389/fnins.2022.824069 (PMC9097855; doi:10.3389/fnins.2022.824069)
Supplement: Supplementary file 1 [file Table_1.DOCX]

***Supplementary Material***

**Supplementary table 1.** Functional annotation for the top SNPs corresponding to each cluster

| **Cluster** | **Chr:Position** | **rsID** | **gwasP** | **beta** | **se** | **Func** | **GenomicLocus** | **Closest gene** | **MAF** |  |
| --- | --- | --- | --- | --- | --- | --- | --- | --- | --- | --- |
|  |  |  |  |  |  |  |  |  |  |  |
| **C1** | **3:130634407** | rs150026771 | 7.03E-08 | 0.0341269 | 0.0062758 | ncRNA_intronic | 91 | ATP2C1:RP11-39E3.3 | 0.008387 |  |
|  | **23:36916369** | rs28688016 | 1.07E-07 | 0.0126073 | 0.0023519 | intergenic | 429 | BX842568.1 | 0.008187 |  |
|  | **13:66510148** | rs59894521 | 1.22E-07 | 0.0114887 | 0.0021531 | intergenic | 337 | MIR548X2 | 0.02436 |  |
| **C3** | **13:51624167** | rs12427819 | 3.49E-08 | 0.004102 | 0.0007371 | ncRNA_intronic | 8 | GUCY1B2 | 0.07548 |  |
|  | **16:75394148** | rs4888388 | 2.94E-07 | -0.001526 | 0.0002953 | intronic | 10 | CFDP1 | 0.499 |  |
|  | **23:131260276** | rs7880476 | 7.99E-07 | -0.001351 | 0.0002717 | intronic | 11 | FRMD7 | 0.4595 |  |
| **C7** | **1:64886612** | rs116202570 | 1.54E-08 | -0.022676 | 0.0039701 | intergenic | 1 | RNU7-62P | 0.01597 |  |
|  | **12:113264090** | rs138291276 | 3.46E-07 | -0.016172 | 0.0031482 | intronic | 31 | RPH3A | 0.007588 |  |
|  | **12:44400475** | rs149212121 | 7.53E-07 | -0.034179 | 0.0068577 | intronic | 30 | TMEM117 | 0.01338 |  |
| **C8** | **23:96450775** | rs5921578 | 2.61E-08 | -0.004879 | 0.000869 | intronic | 82 | DIAPH2 | 0.1883 |  |
|  | **3:41023001** | rs145884040 | 9.33E-09 | -0.019857 | 0.0034222 | intergenic | 11 | RP11-520A21.1 | 0.006789 |  |
|  | **6:154249852** | rs114047728 | 1.04E-08 | -0.034692 | 0.0060014 | intergenic | 41 | HMGB3P19 | 0.01098 |  |
